# Co-inhibition of EGFR and IGF1R synergistically impacts therapeutically on adrenocortical carcinoma

**DOI:** 10.18632/oncotarget.8827

**Published:** 2016-04-18

**Authors:** Lieyu Xu, Yicheng Qi, Yunze Xu, Jianpo Lian, Xiaojing Wang, Guang Ning, Weiqing Wang, Yu Zhu

**Affiliations:** ^1^ Department of Urology, Ruijin Hospital, School of Medicine, Shanghai Jiaotong University, Shanghai, China; ^2^ Department of Endocrinology, Clinical Center of Shanghai Endocrine and Metabolic Diseases, Ruijin Hospital, School of Medicine, Shanghai Jiaotong University, Shanghai, China; ^3^ Department of Urology, Ren Ji Hospital, School of Medicine, Shanghai Jiaotong University, Shanghai, China

**Keywords:** adrenocortical carcinoma, EGFR, IGF1R, coinhibition therapy, crosstalk

## Abstract

**Purpose:**

Adrenocortical carcinoma (ACC) is a rare tumor with very poor prognosis and no effective treatment. The aim of this study was to explore a novel therapy co-targeting EGFR and IGF1R *in vitro and vivo*.

**Methods:**

The expression of EGFR and IGF1R were evaluated in a series of adrenocortical tumors by immunohistochemistry. Cell viability of ACC cell lines H295R and SW13 were determined by MTT assay after treatment with the combination of EGFR inhibitor Erlotinib and IGF1R inhibitor NVP-AEW541. Apoptosis was assessed by flow cytometry. The mechanism within intracellular signaling pathways was analyzed by Western blot. Mice bearing human ACC xenografts were treated with Erlotinib and NVP-AEW541, and the effects on tumour growth were assessed.

**Results:**

Our results show a significant over-expression of EGFR (66.67%) and IGF1R (80.0%) in ACC. Besides, the co-overexpression of EGFR and IGF1R was seen in 8/15 ACCs, as compared with ACAs (*P*<0.05). Erlotinib and NVP-AEW541 significantly inhibited cell viability and induced apoptosis by blocking phosphorylation of MEK/ERK and AKT, respectively. Meanwhile, we found that single inhibition of IGF1R induced compensatory activation of MEK/ERK, leading to sustained activation of mTOR, which represent as aggregation of EGFR and IGF1R downstream components. More importantly, the combination of Erlotinib and NVP-AEW541 enhances anti-tumour efficacy compared to treatment with either agent alone or to untreated control *in vitro and vivo*.

**Conclusions:**

In conclusion, coinhibition therapy targeting EGFR and IGF1R may be considerable for treatment of ACC in the future.

## INTRODUCTION

Adrenocortical carcinoma (ACC) is a rare endocrine malignancy, with annual prevalence of only 0.5 to 2 cases per million people [1]. It is extremely aggressive and nearly half newly diagnosed are advanced diseases, whose 5-year survival rate is often less than 15%. Therapeutic options for this rarity are limited, and often could not improve overall survival, some of those even presented with adverse effects [2]. Nowadays, the only approved first-line drug for metastatic ACC is mitotane, also well known for its narrow therapeutic window and toxicity on gastro-intestinal tract and nervous system [3].

During last decades, targeted therapies have been applied to ACC. The abnormal activation of Insulin-like growth factor 1 receptor (IGF1R) signaling has been demonstrated to be associated with cell proliferation, apoptosis, angiogenesis and resistance in several cancers [4, 5]. The Insulin-growth factor 2(IGF2)-IGF1R pathway has also been reported to play a significant role in the tumorigenesis of ACC [6,7]. IGF system could impact on the downstream intracellular signaling, leading to activation of PI3K/AKT/mTOR and/or RAS/RAF/MAPK pathways [8]. As a result, the IGF-1R system has emerged as a promising target, and several IGF-1R inhibitors and anti-IGF1R mAbs have been investigated. It has been demonstrated that IGF1R inhibitors could inhibit proliferation and promote apoptosis, even with alleviation of mitotane-associated cytotoxicity in ACC cell lines [6, 9]. However, clinical trials targeting IGF1R, such as IMC-A12, OSI-906 and figitumumab, all showed comparatively poor efficiency [10–12]. Therefore, all these results demonstrated that single inhibition of IGF1R is not sufficient to improve overall survival, owning to heterogeneity of ACC, and led our insight into alternative combination therapy.

For several tumors, EGFR has been reported to play pivotal role in tumorigenesis [13,14]. It has been reported that EGFR and IGF1R may cross-talk through heterodimers directly [15–17]. The EGFR has also been reported dysregulation in ACC [18,19]. However, whether crosstalk between EGFR and IGF1R in ACC exists remains unclear. In this study, we firstly evaluated the expression of IGF1R, EGFR and its signaling protein expression in human adrenocortical tumors, then investigated the crosstalk between EGFR and IGF1R pathway, and confirmed the therapeutic effect of co-inhibition of EGFR and IGF1R in ACC.

## RESULTS

### The expression of EGFR, IGF1R, p-mTOR, and p-ERK in ACT

**Figure 1:**
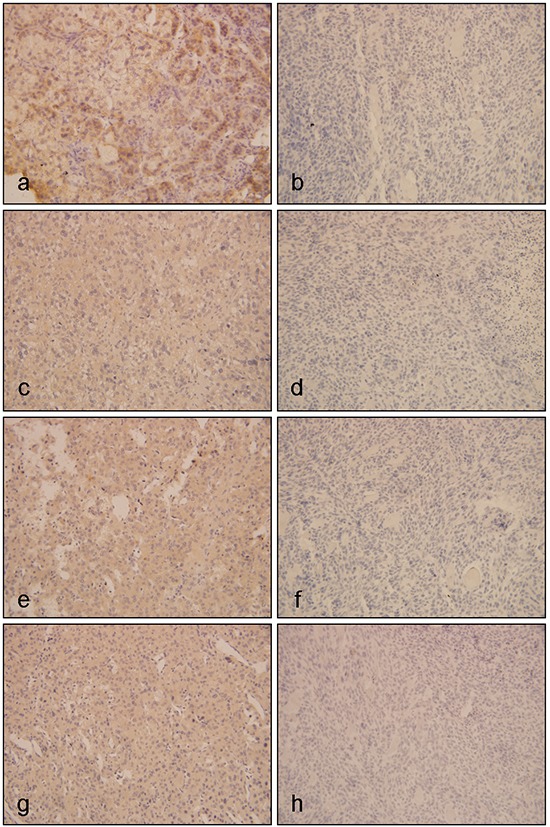
Immunohistochemistical results of EGFR, IGF1R and its downstream proteins in ACTs Figure **a and b.** represented expressions of EGFR in ACTs, of which a was positive expression of EGFR in ACC, and b was negative expression of EGFR in ACA; **c and d.** represented expressions of IGF1R in ACTs, of which c was positive expression of IGF1R in ACC, and d was negative expression of IGF1R in ACA; **e and f.** represented expressions of p-ERK in ACTs, of which e was positive expression of p-ERK in ACC, and f was negative expression of p-ERK in ACA; **g and h.** represented expression of p-mTOR in ACTs, of which g was positive expression of p-mTOR in ACC, and h was negative expression of p-mTOR in ACA. (Original magnification x400)

We firstly detected the expression of EGFR, IGF1R, p-mTOR and p-ERK in a series of ACC tissues and ACA tissues by IHC. As shown in Figure 1, the frequency of positivity of EGFR in two groups was 10/15 (66.67%) and 6/20 (30.0%, *P*=0.044, Figure 1a-1b). IGF1R positive expression was seen in 80.0% (12/15) of ACC, which was 35.0% (7/20) for ACA (*P*=0.016, Figure 1c-1d). Meanwhile, eight out of 15 ACCs (53.33%) were stained positive for both EGFR and IGF1R, which was only 4/20 for ACA. The negative staining for both proteins was 13.33% (*n*=2) in ACCs, compared to 50% at the benign group. Among the 15 ACC cases, neither EGFR nor IGF1R expression was associated with clinical characteristics, including age, ENSAT stage and Weiss score (Supplementary Table S3). Furthermore, the frequency of positivity of p-ERK was 11/15 (73.3%) in ACCs, and 7/20 (35.0%) in ACAs (*P*=0.041, Figure 1e-1f). Positive staining for p-mTOR was observed in 60.0% of the ACC group and 20.0% of the benign group, the difference of p-mTOR expression between ACA and ACC was statistically significant (*P*<0.001).

### The effect of single inhibitor on the downstream signaling pathway of EGFR and IGF1R

**Figure 2:**
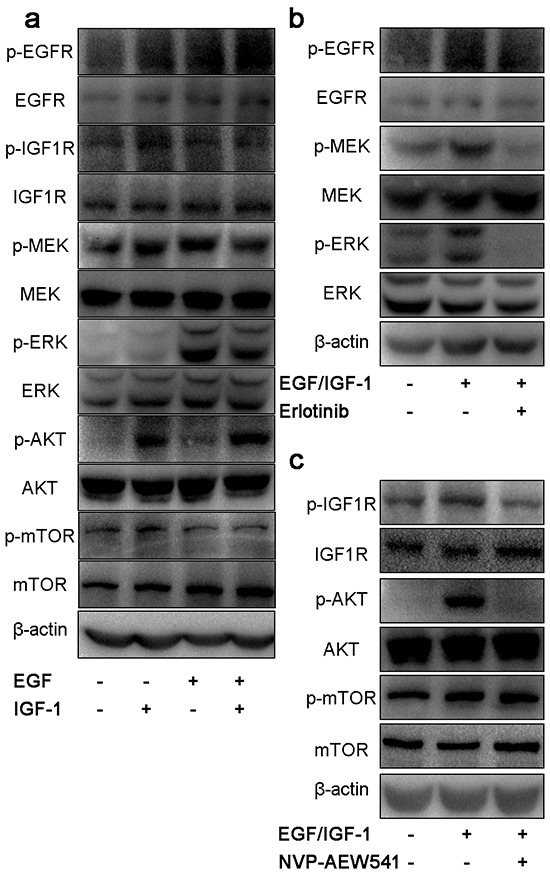
Effect of single inhibitor on EGFR and IGF1R downstream signaling pathways in SW13 cells SW13 cells were grown in 10% FBS followed by 24h incubation in 0.1% FBS growth medium. After that, cells were stimulated with 100μg/L of EGF, 50μg/L of IGF-1 or both for 30 min **a.** treated with 20μM erlotinib **b.** and 2μM NVP-AEW541 **c.** for 24h. Then cells were lysed, proteins were separated by SDS-PAGE, transferred onto PVDF membranes and probed with antibodies. Each experiment was repeated in triplicate.

Firstly, we examined the effect of EGF or IGF-1 on intracellular signaling pathways using ACC cell line SW13. Our result showed that EGF could induce the activation of MEK/ERK, and IGF-1 induced the activation of AKT/mTOR (Figure 2a). EGF-stimulated phosphorylation of EGFR and downstream MEK and ERK were significantly abolished by Erlotinib. NVP-AEW541 could suppress IGF-1 stimulated phosphorylation of IGF1R and downstream AKT, but the levels of p-mTOR was unchanged (Figure 2b and Figure 2c).

### IGF1R inhibition induces compensatory activation of ERK

**Figure 3:**
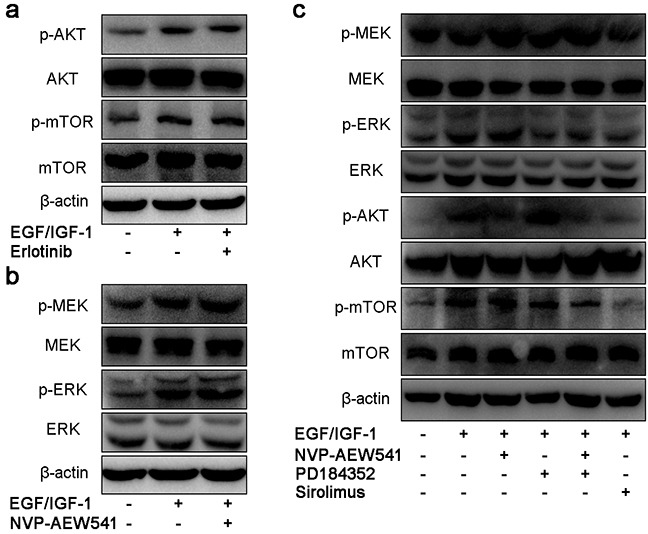
Single blockage of IGF1R induced compensatory activation of MEK/ERK, leading to sustained activation of mTOR in SW13 cells SW13 cells were pretreated as previous described, following 24h incubation with inhibitors or 30 min of growth factors, then protein were assessed by SDS-PAGE, transferred onto membranes and probed with antibodies. The concentration of erlotinib **a.** NVP-AEW541 **b.** PD184352 and Sirolimus **c.** was 20μM, 2μM, 200nM and 50nM, which could inhibit cell viability with little cytotoxic effects. Each experiment was repeated more than three times.

Because NVP-AEW541 could not suppress the activation of mTOR pathway, we aimed to find whether it is the cross-talk effect between EGFR and IGF1R. As shown in Figure 3a, Erlotinib could not regulate IGF-mediated intracellular pathway. However, single inhibition of IGF1R by NVP-AEW541 could induce compensatory activation of ERK (Figure 3b). To further assess the association of ERK and mTOR signaling, we used PD184352 and NVP-AEW541 in order to dual inhibition of ERK and IGF1R, and the results showed that co-inhibition of ERK and IGF1R could downregulated the level of p-mTOR (Figure 3c). Thus, we concluded that the compensatory activation of ERK could lead to the sustained activation of mTOR pathway.

### Combinational therapy inhibit EGFR and IGF1R downstream signaling pathway

**Figure 4:**
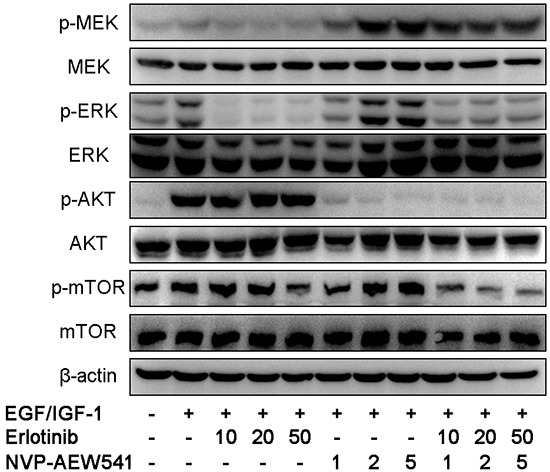
Combinational therapy with erlotinib and NVP-AEW541 could inhibit EGFR and IGF1R downstream signaling pathways in SW13 cells After 24h incubation with inhibitors or with growth factors for 30 min before harvest, cells were lysed and protein expressions were assessed by western blotting. The concentration of erlotinib was 10 to 50 μM, of which was 1 to 5 μM for NVP-AEW541, which could inhibit cell growth with little cytotoxic effects, and the combinational therapy with concentration of inhibitors accordingly. Experiments were repeated more than three times.

To suppress the crosstalk, we investigated the effect of coinhibition therapy by Erlotinib and NVP-AEW541 on the MEK/ERK and AKT/mTOR signaling pathway. Our results showed that coinhibition therapy could simultaneously block downstream signaling components of EGFR and IGF1R pathways, including p-mTOR (Figure 4).

### Combinational therapy could synergistically inhibit cell viability and induce apoptosis in ACC cell lines

**Figure 5:**
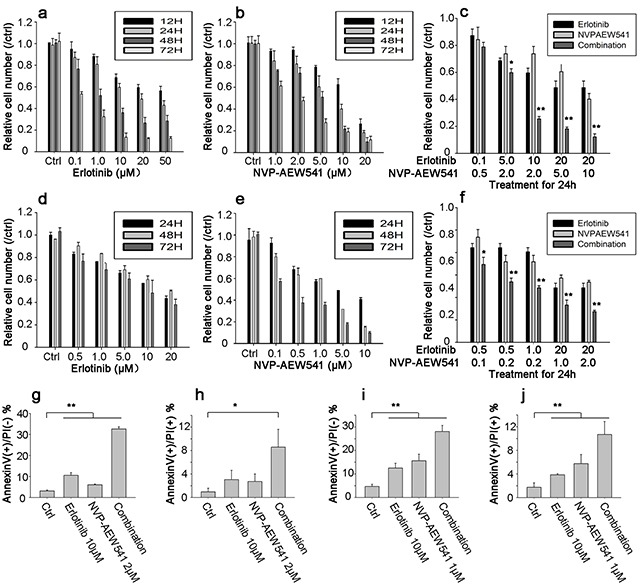
Combinational therapy synergistically inhibited cell viability and induced apoptosis in SW13 and H295R cells Cells were grown in 10% FBS followed by 24h incubation in 0.1% FBS medium. After that, inhibitors were incubated with concentration for 12 to 72h in SW13 cell line **a-c.** and 24 to 72h in H295R cell **d-f.** Cell viability was calculated as percentage of control, and each value was repeated in triplicate (comparison between combinational group and single inhibitor group, *: *P* <0.05, **: *P*<0.01). Figure g-j represented bar figures of early apoptosis and late apoptosis percentages in SW13 **g-h.** and H295R **i-j.** in which AnnexinV+/PI- axis (g and i) represented figures of early apoptosis, and AnnexinV+/PI+ for late apoptosis or death (h and j). Comparison between treatment and control group, *: *P*<0.05, **: *P*<0.01).

MTT assay was performed to examine the effect of Erlotinib, NVP-AEW541 and combinational therapy on relative cell number of ACC cell lines. The results of MTT assay showed that NVP-AEW541 induced a dose and time-dependent decrease of cell viability in SW13 and H295R cells, with IC50 values of 1.06 μM and 0.26 μM at 72 h of treatment, respectively (Figure 5b and 5e). Meanwhile, Erlotinib determined a cytotoxic effect in SW13 and H295R cells with IC50 value of 0.23μM and 3.43μM (at 72 h of treatment), respectively (Figure 5a and 5d). The combination of Erlotinib and NVP-AEW541 showed a significant synergistic anti-proliferative effect on SW13 cells (CI = 0.58±0.23, range: 0.24-0.85) and H295R cells (CI =0.20±0.05, range: 0.12-0.26; Figure 5c and 5f), in which a CI <0.9 indicate synergism.

Additionally, Annexin V-FITC/PI double staining results showed that increasing concentration of Erlotinib or NVP-AEW541 could induce early apoptosis and lead to cell death in both cell lines. The early apoptosis percentages by Erlotinib were 10.54±1.30 and 12.55±1.99% for SW13 and H295R cells, and that of NVP-AEW541 were 6.07±0.31 and 15.63±2.79%, respectively (Figure 5g and 5i). Furthermore, the combination of Erlotinib and NVP-AEW541 showed a significant synergistic induction of early apoptosis, which was 32.62±1.05 and 28.05±2.61 % for SW13 and H295R (*P*<0.01; Figure 5h and 5j). All these results indicated that the co-inhibition therapy could synergistically inhibit cell viability and induce apoptosis.

### Combinational regimen synergistically inhibited tumor growth of SW13 cell xenograft

**Figure 6:**
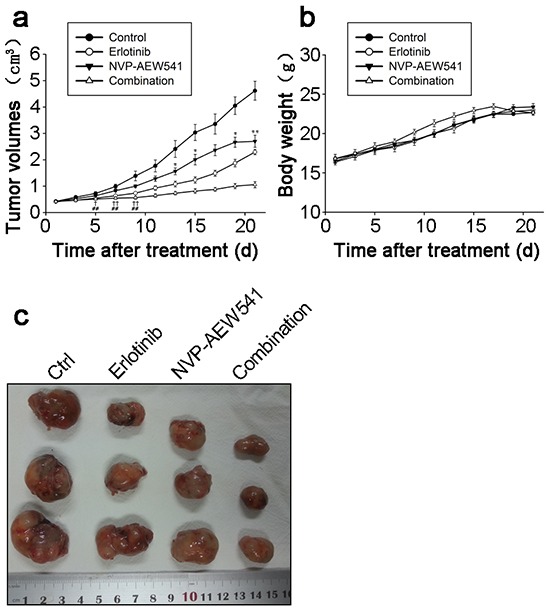
Combinational regimen synergistically inhibited tumor growth of xenograft These nude mice were transplanted with SW13 cells, and assigned to four groups randomly after grown to appropriate volume, which were control (25mML(+)-tartaric acid p.o.), erlotinib (20mg/kg i.p.), NVP-AEW541 (20mg/kg p.o.) and combinational group (erlotinib and NVP-AEW541) for 21 days, each containing 8 mice. The tumor volumes were represented in Figure 6a. The comparison between NVP-AEW541 and control group, *: *P*<0.05; **: *P*<0.01. The comparison between erlotinib and control group, †: *P*<0.05; ††: *P*<0.01. The comparison between combinational and control group,^#^: *P*<0.05; ^##^: *P*<0.01. The gross appearances of tumors were represented in Figure 6b and body weights were represented in Figure 6c.

Given the synergistically inhibitory effects of Erlotinib and NVP-AEW541 on ACC growth *in vitro*, it is believed that combinational regimen has the potential to be highly effective in treating ACC *in vivo*. Thus, we sought to determine the inhibition effect of Erlotinib and NVP-AEW541 on the tumor growth of SW13 cell xenograft. After 21 days, the xenograft tumors of control group grew to average volume of 4.62±0.88 cm^3^ from the beginning 0.42±0.07 cm^3^. As shown in Figure 6a, Erlotinib delayed SW13 cell xenograft tumor growth since day 5 (0.55±0.11 vs. 0.72±0.16 cm^3^, *P*=0.048), and NVP-AEW541 inhibited the xenograft tumor growth since day 13 (1.56±0.32 vs. 2.41±0.78 cm^3^, *P*=0.034). Furthermore, the combination of Erlotinib and NVP-AEW541 showed a significant synergistic inhibition of xenograft tumor growth since day 5 (*P*<0.01), with average volume at 1.06±0.29 cm^3^.

Moreover, gross appearances of xenograft tumors from the combination of Erlotinib and NVP-AEW541 and other three groups were presented at Figure 6b. At the end of experiments, the tumors were isolated and weighted. Compared with control group, the mean tumor weight was significantly less in the combination of Erlotinib and NVP-AEW541 group without affecting body weight obviously (Figure 6c).

## DISCUSSION

ACC is a rare but progressive malignancy, with a bimodal distribution at age of 40 to 50 years and children [24]. Few therapeutic options are nowadays available owning to its rarity and aggressive [1]. With the exception of radical resection for early ACCs, most patients would only benefit little from adjuvant treatments [2]. It is acknowledged that IGF pathway presented as one of the most frequent alterations in ACC carcinogenesis [25]. IGF2 could then elicit its intracellular effects through IGF1R, which was also over-expressed in most ACCs, consistent with our results. Besides, the activation of EGFR pathway has also been reported in ACC [18–19, 26], which was also validated in our study. Therefore, our results further substantiated the aberrant activation of EGFR and IGF signaling pathway in carcinogenesis of ACC.

According to the constitutive activation of IGF1R and EGFR in ACC, we then studied the effect of single inhibitors on growth of ACC *in vitro*. Consistent with previous reports, blockage of IGF1R by NVP-AEW541 could inhibit cell viability by suppression of AKT protein, and induce moderate apoptosis [6,9]. Meanwhile, we found that NVP-AEW541 delayed slightly tumor growth in SW13 cell xenograft, which was in line with previous reports of H295R and RL251 cells [9]. Additionally, we found that EGFR inhibitor Erlotinib could inhibit cell viability with comparable concentration, and also induce moderate apoptosis. Similarly, single inhibition of EGFR could only delay xenograft tumor growth, but not inhibit xenograft tumor growth. Therefore, we supposed that blockade of single pathway is not effective enough to inhibit carcinogenesis owning to sustained activation of alternative survival pathway, which may serve as salvage for tumorigenesis of ACC.

Furthermore, clinical trials of single target therapy on either these two pathways were proved to be not so optimistic. The therapy by IGF1R inhibitor IMC-A12 and mitotane was then reported to present with limited efficacy, with only one partial response [10]. In another trial by IGF1R inhibitor figitumumab, no objective responses were seen among all 14 refractory ACC patients [11]. Recently, another IGF1R inhibitor OSI-906 was investigated in patients with advanced ACC, eventually the results also revealed little therapeutic effects, in which OSI-906 could not increase overall survival of ACC as compared with placebo group [12]. Meanwhile, single blockage by EGFR inhibitors has also been reported with limited therapeutic activity. Therapeutic effect with erlotinib and gemcitabine has been reported to be disappointed in advanced ACCs, in which only one in ten patients experienced minor response [19]. Another EGFR inhibitor gefitinib was also reported to be with no significant efficacy similarly [2].

Therefore, we hypothesized that there exist the cross-talk between EGFR and IGF1R, leading to the failure of treatment of single targeted drug. Previous studies have reported that cell surface interactions between these two pathways could occur directly by heterodimers, or indirectly by mediation of G-protein coupled receptors within other tumours [15]. On the other way, EGFR signaling pathway could induce expression of insulin receptor substrate in breast cancer cells, which could stimulate IGF pathway [27]. Moreover, another study revealed that activation of IGF1R could lead to recruitment and activation of c-Src, which induced the phosphorylation of EGFR [28]. In this study, we found that single inhibition of IGF1R signaling pathway by NVP-AEW541 could lead to compensatory activation upregulation of p-ERK in ACC. Therefore, we demonstrated that tumor may compensate strategies targeting IGF1R by activating alternative EGFR downstream signaling pathway.

In this study, it was surprisingly seen that single inhibition of IGF1R by NVP-AEW541 could not suppress the expression of mTOR, which is a serine/threonine kinase involved in the cell proliferation, apoptosis, angiogenesis, metabolism, and protein synthesis [29]. This could be explained by compensatory activation of ERK pathway, which was reported to further activate mTOR by phosphorylation of TSC2 and Raptor or by AMPK pathway [30,31]. In our study, dual inhibition of ERK and IGF1R could significantly suppress the phosphorylation of mTOR, which further confirm the interaction between ERK and mTOR in ACC. Moreover, we found that combinational therapy targeting both EGFR and IGF1R also significantly inhibited the expression of p-mTOR, owing to abolishment of the upregulation of ERK pathway.

Previously, coinhibition therapies targeting EGFR and IGF1R have been reported to be synergistically effective in other solid tumours [32–34]. Furthermore, a phase II study of anti-IGF1R mAb MK-0646 in combination with anti-EGFR mAb cetuximab and irinotecan has been reported to be effective and tolerable [35]. Additionally, it has been reported that this combinational regimen would be well tolerated owning to lack of duplicate toxicities between inhibitors of IGF1R and EGFR [36]. However, at this point, no studies have examined the role of co-inhibition of EGFR and IGF1R in the treatment of ACC. To abolish the cross-talk between EGFR and IGF1R signaling pathway, we investigated the effect of co-inhibition of EGFR and IGF1R on ACC cell model for the first time. We found that co-inhibition of EGFR and IGF1R could synergistically inhibit cell viability and induce apoptosis potently in two ACC cell lines. Moreover, the combination of Erlotinib and NVP-AEW541 showed greater inhibition of tumour growth than either agent alone in xenograft tumor growth. Therefore, it could be presumed that coinhibition of EGFR and IGF1R would be promising therapy for patients with ACC.

## CONCLUSIONS

In this study, we found the cross talk between EGFR and IGF1R downstream signaling pathways in ACC, in which inhibition of IGF1R could induce compensatory activation of ERK pathway. Furthermore, combinational therapy targeting EGFR and IGF1R could synergistically inhibit cell viability, induce apoptosis *in vitro* and inhibit tumor xenografts growth *in vivo*. In conclusion, we demonstrated that coinhibition therapy targeting EGFR and IGF1R would be considerable for treatment of ACCs in the future.

## MATERIALS AND METHODS

### Patients and tissue samples

The patients diagnosed with adrenocortical tumor (ACT), were recruited between January 1996 and October 2012 at our institution. Apart from formalin-fixed and paraffin-embedded tumor tissues of ACCs, there were also specimens of adrenocortical adenoma (ACA). For all cases, the clinical, pathologic, and follow-up data were collected after ethical approval from local review board. The diagnosis of ACC were based on the evidence of clinical symptoms, endocrine evaluations, imagine examinations, and eventually the pathological diagnosis by the Weiss's criteria with score ≥ 3. All of the patients' characteristics were shown in Table 1 (detailed listed in Supplementary Table S1).

**Table 1:** Clinicopathologic characteristics of patients in this study

Clinical characteristic	ACCs	ACAs
Number	15	20
Gender		
Female	8	9
Male	7	11
Age at diagnostic (year)	50.28±10.36	45.38±9.55
(range)	(40-70)	(39-67)
Tumor size (cm)	8.62±2.37	4.20±1.25
<10 cm	12	20
>10 cm	3	0
Tumor location		
Left	7	12
Right	8	8
Weiss score	5.67±1.40	1.30±0.47
ENSAT Stage		
I	2	
II	4	
III	7	
IV	2	
Previous therapies		
Surgery	15	20
Adjuvant therapy	10	0
Follow-up (years)	4.26 (0.5-8)	10.21(7-16)

### Immunohistochemistry

Serial 4-μm-thick paraffin sections cut from tissue blocks of were processed, dewaxed in xylene, rehydrated by serial concentrations of ethanol, and then rinsed in PBS followed by 3% H_2_O_2_. After heated in a microwave for 15 min, the sections were incubated with 10% normal goat serum at room temperature for 10 min. Sections were incubated with polyclonal rabbit antihuman EGFR, IGF1R, p-mTOR and p-ERK1/2 antibody diluted to 1:150 for 12 h at 4°C. The slides were then followed by a PBS wash, incubated by anti-mouse Envision™ kit for 30 min, and developed in diaminobenzidine substrate. The sections were counter-stained in hematoxylin for 2 min and then dehydrated in ethanol and xylene. Sections were re-prepared by Envision immunohistochemical staining. The positive controls were breast cancer and prostate cancer with positive expressions of EGFR and IGF1R, and PBS was set as negative control.

To confirm reality, all slides were analyzed independently by 2 or more pathologists, who were blinded to the subtypes. Positive staining was characterized by purple-brown granules located diffusely in the cell cytoplasm and/or nuclear. A semi-quantitative scoring system was assessed to evaluate the staining of relevant proteins, according to the percentage of positive staining cells, including 0 (<5%), 1 (5-29%), 2 (30-50%), 3 (>50%), and the intensity of staining, including 0(no), 1(weak), 2(moderate), 3(strong). After combining these two variables, a total score of more than 3 was considered positive and a score of 3 or less was considered negative, as previously reported [20].

### Cell lines and culture situation

The ACC cell lines H295R and SW13 were obtained from the American Type Culture Collection (Manassas, VA, USA). They were cultured in 60 cm^2^ dishes at 37°C in a humidified incubator at 5% CO_2_. The medium for H295R were consisted of DMEM/F12 (Gibco, USA), supplemented with 2.5% Nu-serum I (Corning, USA), 1% ITS+ Premix (Corning, USA), 1% L-glutamine and 1% penicillin-streptomycin (Gibco, USA). SW13 cells were grown in DMEM medium (Gibco, USA) supplemented with 10% fetal bovine serum and 1% penicillin-streptomycin.

### Drugs and reagents

The EGFR inhibitor Erlotinib, IGF1R inhibitor NVP-AEW541, ERK 1/2 inhibitor PD184352 and mTOR inhibitor Rapamycin (Sirolimus) were all purchased from Selleck Chemicals (California, USA). Drugs were dissolved in DMSO at recommended concentration and stored at −20°C, and diluted in culture medium respectively with <0.1% concentration of DMSO. EGF and IGF1 were purchased from Sino Biological Inc. (Beijing, China), and dissolved in water with final concentration at 100 and 50μg/L, respectively. All primary antibodies used were purchased from Cell Signaling Technology (Boston, USA), which included anti phospho-EGFR^Tyr1068^#3777, anti EGFR#2085, antiphospho-IGF1Rβ^Tyr1316^#6113, antiIGF1Rβ #9750, antiphspho-MEK1/2^Ser217/221^ #9154, anti MEK1/2 #4694, antiphospho-Erk1/2^Thr202/Tyr204^ #8201, antiErk1/2 #9102, antiphospho-Akt^Ser473^ #4060, anti Akt#4691, antiphospho-mTOR^Ser2448^ #5536 and anti mTOR #2983 antibodies.

### Cell viability assay

The effect of Erlotinib and NAP-AEW541 on the viability of ACC cell lines were investigated by MTT assay. Briefly, cells were seeded at a density of 5×10^3^/well in 100 μL culture medium in a 96-well plate. After 24h incubation for SW13 or 72h for H295R, serial concentrations of Erlotinib and NVP-AEW541 were added. At the end of each time point fresh MTT was added to each well with final concentration at 0.5 mg/ml. After incubation for 4h, cells were lysed with 150 μL DMSO for 30min to dissolve the crystals. The absorbance was measured at 450 nm, and percentage of cell viability was calculated relative to control.

A dilution of ratios of drug combination method was used in viability assay to determine whether there was synergy, additivity, or antagonism when two drugs were added. Interaction between drugs were assessed using the combination index (CI) as described [21]. Data from the cell viability assay was analyzed using Calcusyn 2.0 software package (Biosoft, Cambridge, UK) to assess drug-drug interactions. A CI < 0.9 indicates synergy, a CI between 0.9~1.10 represents additive effects and a CI > 1.1 denotes antagonism interactions.

### Apoptosis assay

After treated with inhibitors of different concentration for 24 h, SW13 and H295R cells were harvested and washed. They were double stained with Annexin V and Propidium Iodide (BD, USA) for 10 min by instruction from manufacturer, and detected by flow cytometry.

### Protein extraction and western blot

Cells were then lysed in ice-cold lysis buffer (50mM Tri-HCl pH 6.8, 150mM NaCl, 10mM EDTA, 10mM Na_4_P_2_O_7_, 2mM VO3- 4, 100mM NaF, 1mM β-glycerophophate, 1% NP40, and protease and phosphatase inhibitor cocktail (Roche Applied Science, Penzberg, Germany). Cell lysates containing equal amounts of 20μg protein were then separated by SDS-PAGE (10% gels) and transferred onto polyvinylidene fluoride membranes (Millipore, Billerica, MA). After blocked with 5% nonfat milk, the membranes were incubated overnight at 4°C with indicated primary antibodies (1:1000) and β-actin (1:10,000; MP Biomedicals, Germany). Afterwards, the membranes were then washed three times with TBS-T, containing 0.05% Tween 20 and Tris-buffered saline, incubated with corresponding secondary antibodies at room temperature for 1 hour (Abcam, Cambridge, USA) and washed again. Target protein bands were visualized using the enhanced chemiluminescence method. Western blot experiments were repeated at least three times.

### Mouse xenograft model

Four-week-old female athymic nude mice (Shanghai Institute of Material Medical, China) were used. They were all kept under specific pathogen-free condition, and performed under aseptic condition after ethic approval of our institution. After trypsinization, a total amount of 5×10^6^ SW13 cells were subcutaneous injected into each mouse dissolved in a volume of 100μL culture medium into the left hind flank [22], and implanted cells grew to form tumors after approximately 2 weeks. When the xenograft tumors grew to volume of 300-400 mm^3^, mice were randomized to four groups (8 mice each group): control (25 mM L (+)-tartaric acid), Erlotinib (20 mg/kg i.p.), NVP-AEW541 (p.o. at 20 mg/kg), and the combination of Erlotinib and NVP-AEW541 group [23]. Both Erlotinib and NVP-AEW541 were dissolved in 25 mM L(+)-tartaric acid, and all mice were treated for 3 weeks continuously. Mice were checked weekly, and tumor nodules were measured with a caliper. The formula of tumor volume was calculated as: tumor volume (mm^3^) = length × width^2^× 0.5236. The mice were sacrificed and tumors were excised and weighed at the end.

### Statistical analysis

All of the relevant characteristics were expressed as average ± standard deviation; statistical analyses were performed with SPSS statistical package v.17.0. Difference of measurement data and enumeration data were compared respectively with Student's *t*-test, chi-square test and analysis of variance. Correlation between variables was examined by Spearman chi-square test or Fisher's exact test. All tests were 2-tailed, and *P* value<0.05 was considered statistically significant.

## SUPPLEMENTARY FIGURES AND TABLES



## Abbreviations

ACC: adrenocortical carcinoma
IGF1R: insulin-like growth factor 1 receptor
IGF2: insulin-growth factor 2
EGFR: epidermal growth factor receptor
ACT: adrenocortical tumors
ACA: adrenocortical adenoma
