# Supplementary material for: Co-inhibition of EGFR and IGF1R synergistically impacts therapeutically on adrenocortical carcinoma
Source: Oncotarget. 2016 Apr 18;7(24):36235–46. doi: 10.18632/oncotarget.8827 (PMC5094996; doi:10.18632/oncotarget.8827)
Supplement: Supplementary file 1 [file oncotarget-07-36235-s001.pdf]

Co-inhibition of EGFR and IGF1R synergistically impacts therapeutically on adrenocortical carcinoma

SUPPLEMENTARY FIGURE AND TABLES

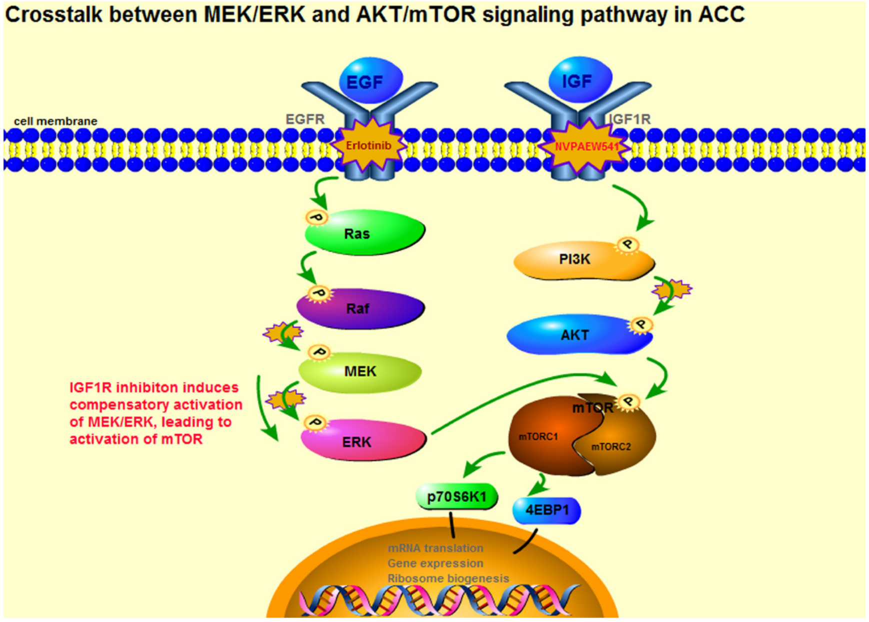

Supplementary Figure S1: Schematic diagram of crosstalk between MEK/ERK and AKT/mTOR in ACC. EGFR inhibitor erlotinib could exert inhibition on phosphorylation of MEK/ERK, and IGF1R inhibitor NVP-AEW541 suppressed levels of phospho-AKT, with inhibition on mTOR. Meanwhile, inhibition by NVP-AEW541 could lead to compensatory activation of MEK/ERK, leading to activation of mTOR pathway. Moreover, combinational therapy could synergistically inhibit mTOR pathway.

Supplementary Table S1: Individual clinical features of 15 ACC patients

Case	Sex	Age*	ENSAT stage	Laterality	Clinical features	Weiss score	EGFR	IGF1R
1	F	64	III	L	Cushing's syndrome, virilization	7	+	+
2	M	57	I	R	Cushing's syndrome	5	+	+
3	F	70	III	L	Cushing's syndrome	4	+	-
4	F	45	II	R	Nonfunctional	6	-	+
5	M	50	III	L	Cushing's syndrome	7	-	+
6	F	42	III	L	Aldosteronism	7	+	+
7	M	51	IV	R	Feminization	8	+	+
8	M	66	I	R	Cushing's syndrome	4	+	-
9	F	43	II	R	Aldosteronism	6	+	+
10	F	42	III	R	Virilization	4	+	+
11	F	41	II	L	Aldosteronism, virilization	4	-	-
12	F	59	III	R	Nonfunctional	7	-	+
13	M	40	III	L	Cushing's syndrome	6	+	+
14	M	42	II	R	Cushing's syndrome	4	-	+
15	M	42	IV	L	Cushing's syndrome	6	+	+

(*: at diagnosis; M: Male; F: Female)

Supplementary Table S2: Summary of immunohistochemistical results of ACT specimens

Groups		Number of positive expression (rate, %)			
	n	EGFR	IGF1R	p-ERK	p-mTOR
ACCs	15	10(66.67%)	12(80.0%)	11(73.33%)	9(60.0%)
ACAs	20	6(30.0%)	7(35.0%)	7(35.0%)	4(20.0%)
P values		0.044	0.016	0.041	0.032

Supplementary Table S3: Clinicopathologic characteristics in relation to expression of EGFR, IGF1R and co-expression of EGFR and IGF1R

Variables		EGFR			IGF1R			Co-expression of EGFR and IGF1R		
	Total	Positive	Negative	P value	Positive	Negative	P value	Dual positive	Other groups	P value
Total; n	15	10	5		12	3		8	7	
Sex; n				1.0*			1.0*			1.0*
Female	8	5	3		6	2		4	4	
Male	7	5	2		6	1		4	3	
Age; year	50.28	51.7	47.4	0.468	48.08	59.0	0.103	47.63	53.29	0.307
Laterality; n										
Left	7	5	2	1.0*	5	2	0.569*	4	3	1.0*
Right	8	5	3		7	1		4	4	
ENSAT stage				0.158*			0.608*			0.378*
I	2	2	0		1	1		1	1	
II	4	1	3		3	1		1	3	
III	7	5	2		6	1		4	3	
IV	2	2	0		2	0		2	0	
Weiss score; mean		5.7	5.6	0.902	4.67	5.92	0.174	6.13	5.14	0.184

(*: Fisher's exact test)
